# Decision landscapes: visualizing mouse-tracking data

**DOI:** 10.1098/rsos.170482

**Published:** 2017-11-08

**Authors:** A. Zgonnikov, A. Aleni, P. T. Piiroinen, D. O'Hora, M. di Bernardo

**Affiliations:** 1 School of Psychology, Statistics and Applied Mathematics, National University of Ireland, Galway, Ireland; 2 School of Mathematics, Statistics and Applied Mathematics, National University of Ireland, Galway, Ireland; 3 Department of Electrical Engineering and Information Technology, University of Naples Federico II, Napoli, Italy; 4 Department of Engineering Mathematics, University of Bristol, Bristol, UK

**Keywords:** decision making, mouse tracking, dynamical systems

## Abstract

Computerized paradigms have enabled gathering rich data on human behaviour, including information on motor execution of a decision, e.g. by tracking mouse cursor trajectories. These trajectories can reveal novel information about ongoing decision processes. As the number and complexity of mouse-tracking studies increase, more sophisticated methods are needed to analyse the decision trajectories. Here, we present a new computational approach to generating decision landscape visualizations based on mouse-tracking data. A decision landscape is an analogue of an energy potential field mathematically derived from the velocity of mouse movement during a decision. Visualized as a three-dimensional surface, it provides a comprehensive overview of decision dynamics. Employing the dynamical systems theory framework, we develop a new method for generating decision landscapes based on arbitrary number of trajectories. This approach not only generates three-dimensional illustration of decision landscapes, but also describes mouse trajectories by a number of interpretable parameters. These parameters characterize dynamics of decisions in more detail compared with conventional measures, and can be compared across experimental conditions, and even across individuals. The decision landscape visualization approach is a novel tool for analysing mouse trajectories during decision execution, which can provide new insights into individual differences in the dynamics of decision making.

## 1. Introduction

Every minute of every day, we make decisions that affect our personal and professional lives, sometimes to a great extent. Despite this continuous practice, our decisions often leave much to be desired. To understand why we make decisions the way we do, decision-making researchers have mainly focused on *what* people choose, by proposing cognitive processes that would give rise to the observed choice outcome distributions. Outcome distributions, however, constitute relatively loose constraints on the possible processes underlying decision making. Consequently, researchers have argued that decision-making theories should be tested on a functional (process) level rather than just on the level of outcome predictions (e.g. [1]). This can be achieved by measuring behavioural activity during the decision-making process to directly assess *how* a choice is made.

**Figure 1.**
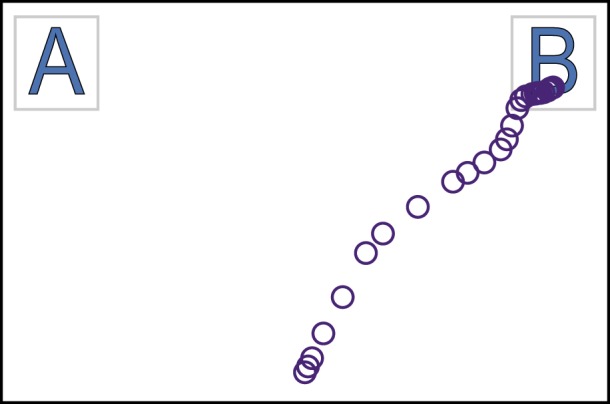
Typical set-up of a mouse-tracking experiment. Circles represent an actual mouse trajectory during binary choice in a learning task [4].

A variety of experimental methods have been used to study the cognitive processes underlying decision making. One class of paradigms, including eye tracking [2] and different variations of the information search paradigm [3], taps attentional processes, trying to answer the question of what information is attended to in the course of a decision. Another strand of research, focused on *hand* or *mouse tracking*, examines how decisions are executed through the motor system. These studies interpret motor output of a decision as a continuous trace of decisional processes. In a typical experiment on mouse tracking, the participant chooses between the two options presented in the corners of a computer screen (figure 1). The dynamics of the response, as expressed in recorded mouse cursor trajectories, can then reveal (post hoc) the degree of competition between the two options during choice.

Mouse (or hand) tracking have been employed to investigate decision-making dynamics in a variety of different domains, e.g. speech processing [5,6], social categorization [7,8], numerical cognition [9,10], intertemporal choice [11,12] and learning [4,13]; see also reviews in [14,15]. The recent development of specialized software for capturing mouse cursor data [16,17] has further increased the amount and complexity of the data generated by mouse-tracking studies.

Response trajectories provide rich continuous data, but the vast majority of available studies use a few relatively simple measures. These include latency measures (initiation or response times), consistency measures (e.g. changes in the *x*-direction (*x*-flips) or sample entropy) and trajectory curvature measures (maximum deviation of the trajectory from an ideal, straight-line trajectory, or area under the curve of difference between actual and ideal trajectories). The analyses of these measures are often highlighted by average mouse trajectories, which may have several important limitations [5,18]. In recent years, further methods have been proposed, including average mouse velocity plots (e.g. [19,20]), trajectory heat maps [11,21], time plots of regression coefficients [21,22], and visualizations based on principal component analysis [23]. However, given the rich spatial and temporal information in response trajectories, it is likely that much potentially important information remains beyond our analytic capacity. More advanced analysis and visualization methods can enable us to get deeper insights from the rich data provided by the mouse-tracking paradigm.

### 1.1. Theoretical foundations of the decision landscape notion

Dynamical accounts of cognition posit that decision making should be treated as a continuous rather than discrete process [24,25]. On the way to our final decision, we must traverse a multitude of intermediate mental states; in other words, we gradually arrive at a decision instead of instantaneously falling into it. On a neural level, this hypothesis is supported by the finding that intermediate states of a decision process are characterized by partially activated neural populations in the dorsal premotor cortex [26,27].

**Figure 2.**
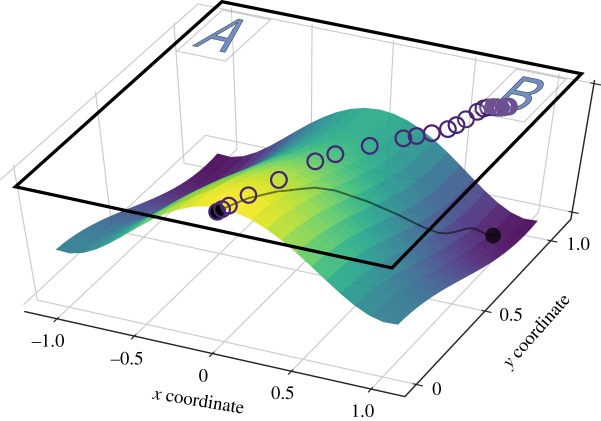
Mouse trajectory from figure 1 and a hypothetical decision landscape driving the decision process. Based in part on fig. 1 in [24].

In line with the foregoing perspective, we assume that the temporal dynamics of a decision can be represented by a trajectory in the multidimensional space of all possible mental states. Some of these states (attractors) are locally stable, so that every decision, although passing through a multitude of intermediate states, eventually gravitates towards one of these attractors. A mouse trajectory can then be treated as a projection of this high-dimensional trajectory onto a two-dimensional space represented by the computer screen [24] (see also [14,23] for an extended discussion of theoretical foundations of the mouse-tracking paradigm). Two attractors (‘pure’ mental states corresponding to finalized decisions) are then mapped onto the locations of the decision outcomes on the screen. The mouse cursor (as a projection of a multidimensional mental state) can then be viewed as a particle moving in an two-attractor force field, much like a marble rolling down one of the two valleys in figure 2. However, up until recently the notion of an attractor landscape driving the decision process remained purely hypothetical. The purpose of this work is to facilitate practical and data-driven applications of this notion.

### 1.2. Current study

Here, we present a computational approach for illustrating mouse-tracking data through three-dimensional visualizations of the *decision landscape*, motivated by recent work in the field [4]. We assume that the decision process, as reflected in a mouse trajectory, and the decision itself are driven by a decision landscape much like the motion of a physical particle in a force field is driven by its potential energy. The parameters defining the shape of the decision landscape can then be tuned to fit a specific decision trajectory (or a set of trajectories). Visualized as a three-dimensional surface, the decision landscape provides a comprehensive overview of motor evolution of decisions. The suggested method can generate illustrations of the decision landscape based on arbitrary number of trajectories and, in addition, can effectively describe each mouse trajectory by a number of interpretable parameters. These parameters, coupled with the generic landscape model, capture the time evolution of decisions in more detail compared with conventional measures. Using previously collected data on a learning task [4], we demonstrate how decision landscape visualizations can be used to compare sets of mouse trajectories between experimental conditions or individual decision makers in a comprehensive and visually appealing way.

## 2. Visualizing decision landscapes: method

**Figure 3.**
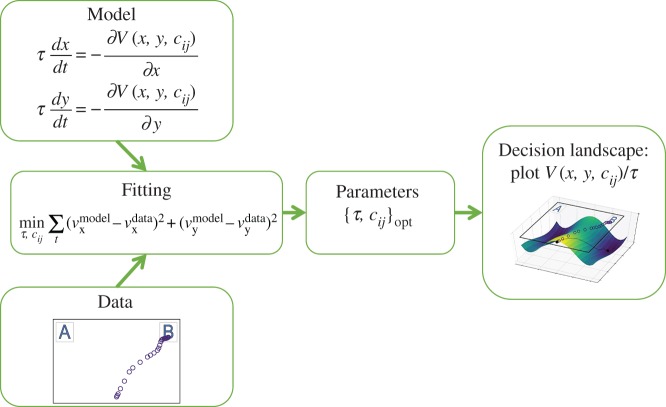
Overview of the decision landscape visualization approach. Mouse data from an experiment are used to fit the parameters of a mathematical model describing a double-well decision landscape.

The proposed method is aimed at reconstructing a three-dimensional decision landscape based on a mouse trajectory of a decision (or a set of trajectories). To do this, we assume that each trajectory can be described by a dynamical system of a specific form, which incorporates a parametrized function describing the shape of the two-attractor landscape. By fitting this dynamical system to a set of trajectories, we obtain specific values of the parameters characterizing these particular trajectories. We can then use these parameters to generate the three-dimensional visualization of the decision landscape characterizing all of the given decisions (see figure 3 for the high-level overview of the method). The source code implementing all the procedures of the method in Python is publicly available [28].

### 2.1. Data requirements and preprocessing

Our method can be used to visualize decision landscapes using the trajectories obtained in a typical mouse-tracking experiment (figure 1). We assume that each decision trajectory starts in the bottom-centre part of the screen and ends in either the top-left or top-right corner. The method can be generalized to the case of more than two choice options; it can also be used with any other experimental paradigm generating simple enough continuous trajectories (for instance, arm reaching [9,29,30], using two-dimensional projections of actual three-dimensional trajectories).

Prior to feeding the experimentally obtained trajectories to the method, we preprocess the trajectories as follows:

— The screen coordinates are rescaled such that each trajectory originates close to (*x*,*y*)=(0,0) and ends near (*x*,*y*)=(−1,1) (left target) or (*x*,*y*)=(1,1) (right target). The reason for this is that the screen size and proportions can differ between experimental set-ups; we thus illustrate the method for spatially normalized trajectories.
— Movement initiation time (the time during which the mouse cursor does not move from the starting position) is disregarded.^1^ The final part of the trajectory (after the mouse has stopped in the response area and its coordinates do not change until the end of the trial) is also discarded from the trajectories.
— Time series describing *x*- and *y*-coordinates of the mouse cursor are resampled so that each trajectory consists of the same number of data points (usually 101).^2^
— For each trajectory, mouse velocities in the *x*- and *y*-directions are computed by taking time derivatives of the *x* and *y* mouse coordinate data. This can be done numerically using finite difference approximations or Savitzky–Golay filters [33].

### 2.2. Model of trajectory dynamics

Without aiming at developing a model *explaining* the dynamics of a decision, we use the simple dynamical system to *describe* the decision trajectory, capturing the high-level features of motion of the mouse cursor. We describe the *x*- and *y*-components of a decision trajectory by a system of differential equations

2.1 $$\begin{array}{rl} \tau \dot{x} & =-\frac{\partial V}{\partial x}\\ \;\text{and}\;\tau \dot{y} & =-\frac{\partial V}{\partial y}, \end{array}\}$$

where *x*=*x*(*t*) and *y*=*y*(*t*) are the positions of the mouse along the *x*- and *y*-coordinates, and $\dot{x}=dx/dt$ and $\dot{y}=dy/dt$ are the time derivatives of *x* and *y*, respectively, *τ*>0 is the time-scale parameter expressed in seconds, *V* (*x*,*y*) is an unknown function describing the decision landscape, which defines the dynamics of the system, and ∂*V* /∂*x* and ∂*V* /∂*y* are partial derivatives of *V* . Our method is not constrained by some particular function *V* (*x*,*y*); here we use one of the simplest possible variants.

We assume that *V* (*x*,*y*) comprises a fixed baseline component *V*_*x*_(*x*)+*V*_*y*_(*y*) and a parametrized component *V*_*xy*_(*x*,*y*):

2.2 $$V(x,y)=V_{x}(x)+V_{y}(y)+V_{xy}(x,y),$$

where *V*_*xy*_(*x*,*y*) can be fitted to the data, and *V*_*x*_(*x*) and *V*_*y*_(*y*) are polynomials chosen in such a way that the two target locations, (−1,1) and (1, 1), are attractors, and the starting location (0, 0) is a repellor of the system (2.1), and thus (with the integration constants set to zero)

2.3 $$V_{x}(x)=\int \frac{\partial V_{x}}{\partial x}\;dx=\int x(x+1)(x-1)\;dx=\frac{x^{4}}{4}-\frac{x^{2}}{2}$$

and

2.4 $$V_{y}(y)=\int \frac{\partial V_{y}}{\partial y}\;dy=\int y(y-1)\;dy=\frac{y^{3}}{3}-\frac{y^{2}}{2}.$$

Having a two-attractor decision landscape as a baseline, we introduce the parametrized polynomial component *V*_*xy*_(*x*,*y*) to be able to account for asymmetry in the landscape and other, more intricate properties of experimental trajectories. Here, also for the reason of simplicity, for *V*_*xy*_(*x*,*y*) we use a polynomial function of *x* and *y*

2.5 $$V_{xy}(x,y)=\sum_{k=2}^{\alpha }\sum_{\begin{array}{c} i,j>0\\ i+j=k \end{array}}\frac{c_{ij}x^{i}y^{j}}{\left(k-1\right)},$$

where the parameter *α*≥2 determines the number of terms in the polynomial, which in turn determines the descriptive ability of the model, and the parameters *c*_*ij*_ are fitted to the mouse data. With increase in *α*, the number of free parameters increases, therefore the fitted values of these parameters may be difficult to interpret for large *α*. Heuristically, we recommend to use the method with *α*=2, 3 or 4, depending on complexity of the trajectories, and taking into account the trade-off between approximation accuracy and interpretability of the parameters.

**Figure 4.**
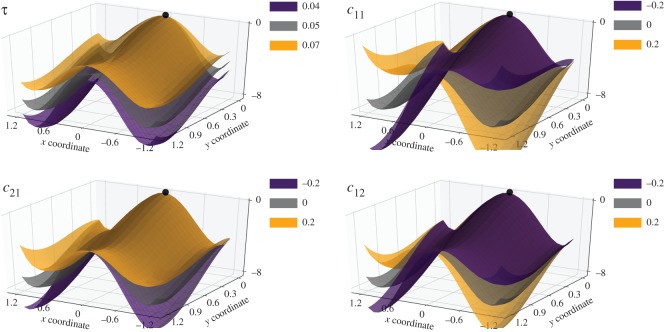
Changes in the baseline decision landscape depending on the parameters of the model for *α*=3. In each panel, all parameters except for the one in the panel title are fixed at the baseline levels *τ*=0.05, *c*_*ij*_=0; the baseline landscape is shown in grey colour. Note that here and in the further figures three-dimensional plots can be rotated differently to illustrate differences in the landscapes.

The effect of the model parameters *τ* and *c*_*ij*_ on the shape of the decision landscape can be analysed independently of the experimental data (figure 4). For any *α*, two parameters, *τ* and *c*_11_, always enter the model. The parameter *τ* affects the characteristic time scale of the system motion: the larger the value of *τ*, the slower the motion of the mouse generated by the model (in both directions).

The only second-order parameter of the model, *c*_11_, is the primary determinant of the asymmetry of the decision landscape. Such asymmetry may be caused, for instance, by strong prevalence of one decision outcome over another. Another possible example would be a situation when trajectories towards one option are consistently faster compared to the trajectories pointing to the other option.

When *α*≥3, two additional parameters enter the model, *c*_21_ and *c*_12_. Their effects are somewhat similar to those of *τ* and *c*_11_, respectively (figure 4); however, these and the other higher-order parameters allow for fine-tuning of the decision landscape to the experimental trajectories.

### 2.3. Fitting the model to one trajectory

For a single experimental trajectory, we aim to find the parameters allowing the model (2.1)–(2.5) to reproduce the dynamics of this trajectory as closely as possible. We can quantify the fitting error in two ways: (i) as a function of the positional difference between the data and the modelled trajectory, or (ii) based on the difference in mouse velocities between the data and the model. The first approach would arguably result in a more accurate approximation of trajectories, but requires substantially more computational effort, as in each step of the fitting algorithm the system of differential equations (2.1) has to be solved.^3^ Here, we focus on the second, velocity-based approach, which is much more efficient in terms of computational resources (although sometimes at the expense of approximation accuracy).

Given an experimental mouse trajectory sampled at *m* time steps and the numerically derived mouse velocity vectors $v_{x}^{\mathrm{data}}$, $v_{y}^{\mathrm{data}}$, we define the fitting error

2.6 $$H(\tau ,c_{ij},v_{x}^{\mathrm{data}},v_{y}^{\mathrm{data}})=\frac{1}{m}\sum_{i=0}^{m}{\left(v_{x}^{\mathrm{model}}(x_{i},y_{i})-v_{x}^{\mathrm{data}}(t_{i})\right)}^{2}+{\left(v_{y}^{\mathrm{model}}(x_{i},y_{i})-v_{y}^{\mathrm{data}}(t_{i})\right)}^{2}.$$

Here, $v_{x,y}^{\mathrm{model}}(x_{i},y_{i})$ are the values of the right-hand side of the system (2.1) (divided by *τ*) computed at each point (*x*_*i*_,*y*_*i*_) along the experimental trajectory

$$\begin{array}{rl} v_{x}^{\mathrm{model}}(x_{i},y_{i}) & =-\frac{1}{\tau }\;\frac{\partial V_{x}}{\partial x}(x_{i},y_{i}),\\ v_{y}^{\mathrm{model}}(x_{i},y_{i}) & =-\frac{1}{\tau }\;\frac{\partial V_{y}}{\partial y}(x_{i},y_{i}). \end{array}$$

These values depend on the parametrization of the model, so the defined error function depends both on the model parameter values and the experimental trajectory.

Using numerical optimization routines (available, e.g. in the Python package scipy.optimize), we can now find the best-fitting values of the model parameters for a given mouse trajectory. Note that the Jacobian of the error functions (2.6) and (2.7) can be analytically derived (see accompanying source code [28]), which enables one to use more efficient and robust optimization algorithms. As an initial approximation for the parameter fitting procedure, the parameters corresponding to the baseline landscape *V*_*x*,*y*_=0 should be used.

### 2.4. Fitting the model to multiple trajectories

Visualizing a decision landscape that would integrate the properties of multiple trials (within a single experimental condition, individual participant or a group of participants) is where the method can prove most useful. To be able to do this, we use the same approach as in the case of a single trial, and minimize the average error across individual trials in the given set. Given the set of *N* trajectories and their velocities $v_{x,n}^{\mathrm{data}}$ and $v_{y,n}^{\mathrm{data}}$ for *n*=1,…,*N*, the fitting error for multiple trajectories is defined by

2.7 $$\hat{H}(\tau ,c_{ij},v_{x,1}^{\mathrm{data}},v_{y,1}^{\mathrm{data}},\ldots ,v_{x,N}^{\mathrm{data}},v_{y,N}^{\mathrm{data}})=\frac{1}{N}\sum_{n=1}^{N}H(\tau ,c_{ij},v_{x,n}^{\mathrm{data}},v_{y,n}^{\mathrm{data}}),$$

where *H* is defined in (2.6).

## 3. Visualizing decision landscapes: examples

We illustrate several potential scenarios of using decision landscapes to visualize mouse-tracking data by applying the technique to previously obtained data on a simple learning task [4] (the data are publicly available [34]). The task consisted of a series of binary choices between abstract symbols, with each symbol yielding either low or high reward (e.g. 5 or 20 points). The goal of the participants was to get as many points as possible throughout a set of 36 trials, which included low versus low, high versus low and high versus high choices. By the end of the experiment, most of the participants successfully learned to choose only the symbols associated with a high reward.

Here, we only consider the part of the data corresponding to high versus low choices (20 per participant), so that the outcome of a decision is either ‘high’ (7, 10 or 20 points depending on experimental condition) or ‘low’ (5 points). Without loss of generality, the data are preprocessed so that the ‘high’ option is mapped to the right-hand corner of the screen (*x*=1), and the ‘low’ option is located in the left-hand corner (*x*=−1). To fit the experimental data, we used the version of the model (2.1)–(2.5) with *α*=3, which has four free parameters. The baseline values of the parameters were set to *τ*=0.05, *c*_*ij*_=0.

### 3.1. Single-trial decision landscapes

**Figure 5.**
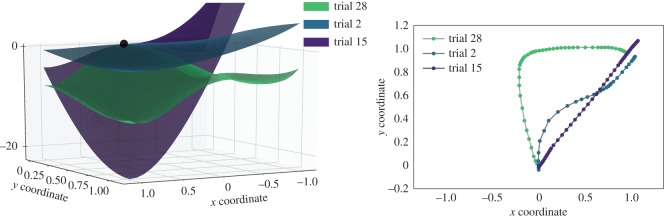
Decision landscapes and trajectories of three representative trials of Participant 90. In all three trials, the subject had chosen the ‘high’ option. In trial 28, the mouse trajectory was moderately fast, but substantially curved towards the unchosen option. Trial 2 was slow and mildly conflicted. In trial 15, the trajectory was fast and close to the straight line. See table 1 for details.

Fitted to a single trial’s mouse trajectory, the decision landscape captures both temporal dynamics and geometry of a trial (figure 5 and table 1). Two key properties of mouse trajectories reflected by the fitted landscapes are: *motion time* (MT), i.e. how long it takes for the cursor to reach the response area once it leaves the starting location, and ‘max-d’, *maximum deviation* from the ideal, straight-line trajectory. Slow trajectories are characterized by shallow landscapes, which indicate weak attraction towards the eventually chosen option (e.g. trial 2 in figure 5). With decreasing MT, the attractor associated with the chosen option becomes stronger, as reflected by the steeper slope of the landscape (trials 28 and 15 in figure 5). However, if the trajectory deviates substantially towards the ‘low’ option, the second, latent attractor emerges in the decision landscape (trial 28), although this option was not chosen. Still, the chosen option attractor appears to be stronger than that of the unchosen option, reflecting the fact that the participant was ‘pulled’ towards it eventually.

**Table 1.** Trajectory measures of three representative trials of Participant 90.

	chosen option	motion time	max-d
trial 28	1 (high)	0.37	0.77
trial 2	1 (high)	0.72	0.23
trial 15	1 (high)	0.15	0.02

### 3.2. Learning

**Figure 6.**
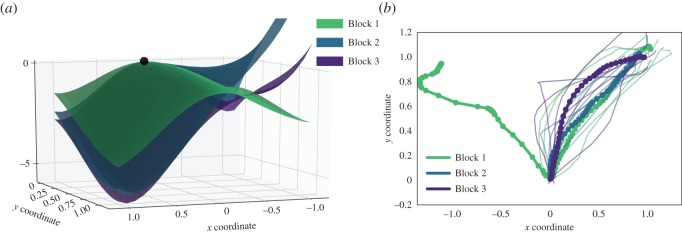
Evolution of decisions of Participant 1334 throughout three consecutive experimental blocks as captured in (*a*) decision landscapes and (*b*) mouse trajectories. In panel (*b*), dotted lines are average trajectories, and faint solid lines are individual trajectories. See table 2 for details.

If the experimental task involves adaptation, the decision landscapes can be used to highlight learning patterns within subjects. By way of example, we pooled all trials of a representative participant into three consecutive blocks, so that the first six ‘high-low’ trials fell into Block 1, the next six into Block 2 and the last eight ‘high-low’ choices formed Block 3. Across blocks, the participant learned to ignore the ‘low’ option (table 2). Figure 6 represents three decision landscapes separately fitted to all trajectories of each block. The decision surface gradually changes from the two-attractor landscape with a more pronounced potential well associated with the ‘high’ attractor to the single-attractor configuration, thereby tracking the learning-induced evolution of preference across blocks. Here, besides the dynamics of the trials, the decision landscape is shaped by the choice distribution: with practice, Participant 1334 learned to ignore the ‘low’ option (in blocks 2 and 3 only the ‘high’ option was selected).

**Table 2.** Mean trajectory measures for three experimental blocks of Participant 1334.

	option	N	motion time	max-d
Block 1	1 (high)	7	0.94	0.18
	−1 (low)	1	1.08	−0.28
Block 2	1 (high)	6	0.72	0.18
	−1 (low)	0	—	—
Block 3	1 (high)	6	0.59	0.31
	−1 (low)	0	—	—

One may note that the decision landscapes suggest that the participant’s aversion to the ‘low’ option has somewhat decreased from Block 2 to Block 3. This is likely to be the consequence of more curved trajectories observed in Block 3, given that the choice distribution and MTs remained similar.

### 3.3. Decision landscapes of individual decision makers

One of the potential applications of the present method is highlighting individual differences between two participants performing the same task. To do this, one can obtain decision landscapes individually for each participant by fitting the landscape model to all trials of that individual simultaneously. In the case of multiple-trajectory fitting, the fitting error is defined as the average error across all trajectories of a given participant (equation (2.7)), so the resulting decision landscape will integrate the information on how often, how fast and with what degree of competition each option was chosen. This will provide a comprehensive overview of the participant’s decisions throughout the experiment.

**Figure 7.**
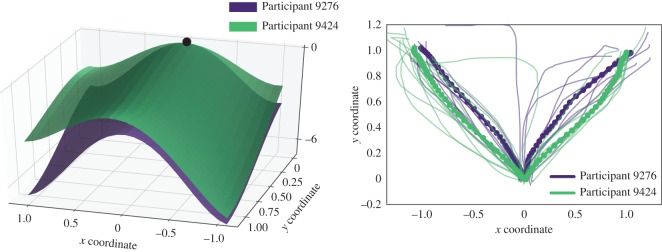
Decision landscapes of two individual participants: Example 1. The shape of the decision landscapes changes depending on the choice distribution produced by the participants. See table 3 for details.

We illustrate this by two representative examples comparing pairs of participants. In the first example (figure 7 and table 3), Participant 9276 was almost equally likely to choose either option, with faster trajectories reaching towards the ‘low’ (left-hand side) option. Participant 9424 had chosen the ‘low’ response more often, and it was chosen on average faster than the ‘high’ option. This difference in choice distributions is reflected in the decision landscapes of the two participants. Particularly, the decision landscape favours the ‘high’ option more in Participant 9276, who chose this option relatively more often than Participant 9424, although the MTs towards this option are similar in the two participants, and despite the fact that trajectories of Participant 9424 towards this option were more direct.

**Table 3.** Mean trajectory measures for Participants 9276 and 9424.

	option	N	motion time	max-d
Participant 9276	1 (high)	8	0.55	0.18
	−1 (low)	9	0.47	0.04
Participant 9424	1 (high)	7	0.53	−0.08
	−1 (low)	13	0.4	−0.07

**Figure 8.**
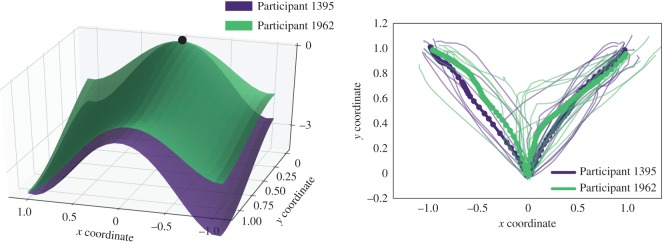
Decision landscapes of two individual participants: Example 2. The choice distributions of the two individuals and corresponding motion times are similar, and the difference in landscapes are mostly due to geometry of the underlying trajectories. See table 4 for details.

The second example highlights the differences in decision landscapes of the participants with similar choice distributions (figure 8 and table 4). Participant 1395 (purple surface) and Participant 1962 (green surface) chose the ‘high’ option in 65% and 70% of the trials, respectively. However, because the trajectories of Participant 1962 towards the ‘low’ choices were more deflected towards the ‘high’ option than those of Participant 1395 (max-d 0.18 versus 0.06), the left-hand side attractor of the green decision landscape is shallower than that of the purple landscape.

**Table 4.** Mean trajectory measures for Participants 1395 and 1962.

	option	N	motion time	max-d
Participant 1395	1 (high)	13	0.72	0.12
	−1 (low)	7	0.61	0.06
Participant 1962	1 (high)	14	0.85	0.20
	−1 (low)	6	0.63	0.18

## 4. Discussion

The decision landscape visualizations provide a comprehensive overview of decision makers’ responses in mouse-tracking experiments. Each visualization integrates the information on (i) the likelihood of each option to be chosen, (ii) the duration of the response and (iii) the degree of competition between the options.

The shape of the decision landscape is influenced (but not completely determined) by the likelihood of choosing each option. If one option was selected much more often than the other, the optimal fit of the landscape might be achieved by effectively ignoring the trajectories towards the less frequent choice, which results in a one-attractor decision landscape. However, in the case of equally likely choices, the best-fit decision landscape must necessarily capture the dynamics of the trajectories in both directions. At the same time, temporal and geometrical aspects of the trajectories also contribute to the shape of the landscape. Slow or very curved trajectories towards an option can lead to decreased attraction of that option as expressed in the decision landscape, even though that option was finally chosen in the end. Consequently, the decision landscape visualizations may aid in interpreting possible paradoxical situations where one option is chosen less frequently, but faster and more directly, and the other option is chosen often, but in a conflicted way.

The parameters of the decision surfaces generated by the method can be used to concisely describe each trajectory (or set of trajectories). Much like coefficients in a Fourier series representation of a periodic function, the parameters of our model define relative contributions of corresponding polynomial terms of the ‘potential energy’ *V* (*x*,*y*) to the overall dynamics of a trial. On the one hand, the polynomial approximation of a trajectory’s mouse velocity is more detailed (and less concise) than the standard discrete measures used in the mouse-tracking literature, namely, response time and maximum deviation. On the other hand, it is less detailed (yet more concise) than the original time series of mouse *x*- and *y*-coordinates.

Traditionally, mouse-tracking data are visualized using mean mouse trajectories in the *x*–*y* plane [16]. This method, being one of the simplest visualization techniques, is easy to interpret: increased attraction towards a competitor option is clearly reflected by a mean trajectory curved towards that option. However, an important limitation of this approach is that, in order to generate mean trajectories, one must either time-normalize trajectories to provide the same number of time steps per trajectory (e.g. by interpolating each trajectory to 101 time steps) or average *x*–*y* locations within temporal bins. In the former case, such mean trajectories allow one to visualize the geometry of the trajectories, but it ignores their temporal dynamics: two geometrically identical mean trajectories of different durations would be indistinguishable in the *x*–*y* plot. In the latter, one can visualize the average temporal dynamics of a set of trajectories, but at the cost of averaging across space, and potentially occluding features of the typical geometry of trajectories. The proposed decision landscape visualization approach addresses this issue by incorporating temporal aspects of the trajectories as well as their geometrical properties.

The first method for visualization of decision landscapes based on mouse tracking data was recently provided by O’Hora *et al.* [4]. Their approach, however, is purely data-driven, and thus requires a large number of trajectories (of order 100) to generate a reliable visualization of a decision surface. By incorporating prior assumptions about decision landscape *V* (*x*,*y*) into a parametrized model, we dramatically reduce the data requirements of our method. As demonstrated above, the method proposed here can be used even with individual trajectories, but can also incorporate an arbitrary number of trajectories. In addition, the decision landscape visualization method reported in [4] is constrained solely to visualization, whereas we, owing to the underlying dynamical model, can extract from each trajectory a set of parameters describing that trajectory. These parameters characterize time evolution of decisions, and can be compared across experimental conditions and even across individual participants.

Even though the current approach can be employed to characterize the average decision landscape for a person or even a condition within an experiment, the utility of the current model reduces as we move further from individual trajectories. As we have demonstrated, ‘average’ landscapes can be meaningfully generated for conditions within a participant. However, when dealing with large numbers of trajectories, the computational costs of our method associated with fitting the dynamical system to the data become too high for practical use, and hence, in this case, methods such as those proposed by O’Hora *et al*. [4] are preferred. It is then possible to apply the approach proposed here to infer the landscape parameters by fitting *V* (*x*,*y*) to these data-driven landscapes; this might facilitate comparison across groups of subjects.

Importantly, the presented version of the method assumes the continuity of trajectories, which is not always the case: in a fraction of experimental trials, participants change their mind during a trial, which is indicated by abrupt shifts in the *x*-direction of a decision trajectory. This happens even in simple perceptual discrimination tasks [29]; depending on the task and the experimental set-up, the frequency of changes of mind can reach 20% (e.g. [35]). The deterministic dynamical model of a decision trajectory used in the present method does not account for such changes of mind. One way to conceptualize such responses is that the landscape of a decision involving abrupt preference shifts should depend on time and, thus, might be described by a non-stationary, possibly stochastic model. Development of a method to infer decision landscapes from such change-of-mind trajectories is an important future research direction.

It is important to note that, though the current visualizations provide a concise way of displaying the most relevant features of mouse cursor movement during a two-choice experiment, they do not constitute a process model of decision making. That is, the attractors at the response locations in the model of a trajectory simply reflect the requirement that a participant chooses one of these responses to complete a decision. The current visualization approach aims to facilitate comparison of mouse data across experimental conditions or consecutive decisions, as we have demonstrated. Moreover, this approach provides a rich outcome space within which to contrast the hypothetical outcomes of proposed process models. For instance, decision-making models that infer differences between earlier processes and later processes within a decision might predict specific changes in these landscapes under certain experimental conditions.

Attractor models have proved useful in understanding *outcomes* of cognitive processes such as categorization [36], risky decision making [37], intertemporal choice [38,39] and perceptual decision making in intermittent motor control [40]. This work is among the first attempts to apply the concepts of dynamical systems theory to process data characterizing decisions. We hope that the proposed decision landscape visualization approach will eventually grow into a new tool for analysing decision trajectories, which will be able to provide new insights into dynamics of decision making.

## Footnotes

1 Recently, a dynamic start procedure has been advocated, where a participant is only presented with the stimuli once they start moving the mouse [11,31,32]. Dynamic start would mitigate the potential problem of meaningful information about the initial stages of decision not being expressed in the mouse trajectory.

2 Resampling to sufficient number of data points is unlikely to distort the relevant spatial information [17].

3 In this case, initial conditions for simulating model trajectories also need to be taken care of. In each experimental trial, the exact starting point of the mouse trajectory is determined by where exactly in the ‘start button’ the participant clicked to begin the trial, which makes this starting point effectively random. However, the system (2.1) is deterministic, so initial conditions fully define its dynamics. Thus, in order to get reasonable fit to the data, one needs to minimize the error function not only over the parameters of the dynamical system, but also over the range of possible initial conditions, which in themselves do not carry any meaningful information. This further adds to computational requirements of this approach.

## Data accessibility

The code implementing the method is publicly available [28], see https://osf.io/5q364/. The data used to illustrate the method were collected previously and are also available online [34], see https://osf.io/ahpv6/.
